# Optimized high-definition tDCS in patients with skull defects and skull plates

**DOI:** 10.3389/fnhum.2023.1239105

**Published:** 2023-10-20

**Authors:** Alexander Guillen, Dennis Q. Truong, Abhishek Datta, Yu Huang

**Affiliations:** ^1^Research and Development, Soterix Medical, Inc, Woodbridge, NJ, United States; ^2^The City College of New York, New York, NY, United States

**Keywords:** transcranial direct current stimulation (tDCS), skull defect, skull plate, tramatic brain injury, computational models and optimization

## Abstract

**Introduction:**

Transcranial direct current stimulation (tDCS) has been shown to benefit patients with brain lesions or traumatic brain injury (TBI). These patients usually have skull defects with different sizes and electrical conductivities. There is very little data in the literature that show how to optimally stimulate these patients with the presence of skull defects.

**Methods:**

Here we leveraged high-resolution (1 mm) realistic head models to explore the best montages targeting right beneath the skull defects with different sizes and conductivities. Specifically, open-source software ROAST was used to solve for the lead field on the publicly available MIDA model. Four different skull defects/plates were modeled with the center above the right primary motor cortex: a larger defect (10 cm diameter) modeled as either titanium or acrylic plate, and a smaller defect (2.5 cm diameter) modeled as either acute state filled with cerebrospinal fluid (CSF) or chronic state with scar tissue. Optimized stimulation with maximal intensity was run using ROAST targeting the right primary motor cortex.

**Results:**

We show that optimized high-definition montages can achieve an average of 0.3 V/m higher stimulation intensities at the target compared to un-optimized montages (M1-SO or 4×1). Large skull defects with titanium or acrylic plates significantly reduce the stimulation intensity by about 80%, while small defects with acute (CSF) or chronic (scar) tissues significantly increase the stimulation intensity by about 200%. Furthermore, one can use M1-SO to achieve almost the same stimulation strength as the optimized montage if the skull has a large defect with titanium plate, and there is no significant difference in stimulation intensity between 4×1 montage and the optimized montage for small skull defects with scar tissue.

**Discussion:**

Based on this work, future modeling studies leveraging individual anatomy of skull defects may help guide tDCS practice on patients with skull defects and skull plates.

## Introduction

As an emerging neuromodulation technique, transcranial direct current stimulation (tDCS) has been shown to have therapeutic effects for a wide range of neurological disorders such as major depression (Bikson et al., 2008), epilepsy (Fregni et al., 2006b; Auvichayapat et al., 2013), Parkinson’s disease (Fregni et al., 2006a), chronic pain (Fregni et al., 2007), and stroke (Meinzer et al., 2016). It is shown that tDCS has the potential to promote motor recovery and improve cognitive functions after traumatic brain injury (TBI) (Kim et al., 2019; Schwertfeger et al., 2023; Ziesel et al., 2023). High-definition (HD) tDCS leverages several small disc electrodes (~6 mm radius) to achieve better focality compared to conventional pad electrodes (Datta et al., 2009). We have previously developed algorithms to optimally guide electrode placement so that a specific brain region can be stimulated with HD-tDCS with either maximal intensity or maximal focality (Dmochowski et al., 2011; Huang et al., 2018). However, all these studies are based on intact skulls. Skull defects and use of skull plates can significantly alter the injected electric current, as shown in previous computational studies (Datta et al., 2010; Sun et al., 2021). To the best of our knowledge, there is still no data reported in the literature that shows if we can efficiently stimulate brain regions below the skull defects or skull plates by optimizing the electrode montages. This is important for patients with TBI as they usually have defects in their skull (also known as the decompressive craniectomy), and tDCS has shown benefits to recovery after TBI (Kim et al., 2019; Schwertfeger et al., 2023; Ziesel et al., 2023). In this study, we aim to computationally investigate how skull defects or plates affect the current flow induced by optimized HD-tDCS. Specifically, we built a realistic, high-resolution computational model following previous methodology (Huang et al., 2019). To find out how different sizes and electrical conductivities of skull defects / plates affect the patterns of current flow, we altered the original model of normal anatomy into four variants that modeled a larger and a smaller skull defect with different conductivities. As the most common locations of the skull defect are unilateral with an opening on the left or right hemisphere (Fatima et al., 2019; Lambride et al., 2020), we modeled the skull defect above the right primary motor cortex. We then performed optimized HD-tDCS (Dmochowski et al., 2011) targeting the right primary motor cortex and compared the achieved electric field at the target with those from an intact skull anatomy. We found that optimization always increases the stimulation at the target below the skull defects. Large skull defects reduce the stimulation intensity while small defects increase the intensity. We hope that our results will provide some general guidelines for future tDCS on patients with skull defects and skull plates.

## Methods

### Construction of head and skull lesion models

A high-resolution (0.5 mm) head model publicly available at the IT’IS Foundation known as MIDA (Multimodal Imaging-Based Detailed Anatomical Model, Iacono et al., 2015) was used in this study. The original MIDA model has segmentation for 153 brain structures. As the goal of this work is to evaluate how skull defects affect optimized HD-tDCS, we are interested in a head model that includes the major head tissues. Therefore, we merged most of these structures into six tissue types: white matter, gray matter, cerebrospinal fluid (CSF), skull, scalp, and air cavities. This was done in ScanIP (Simpleware Ltd., Exeter, UK). The model was also downsampled to 1 mm resolution for faster speed in computing the lead field (see Section of “Optimized HD-tDCS”).

Patients with a large skull defect (up to a diameter of 10 cm) that can be associated with decompressive craniectomy (Guo et al., 2022) usually have a skull plate implanted for cosmetic purposes and to also protect against external trauma, as the original skull cannot be placed back (Sekhar and Fessler, 2016). A small skull defect (diameter of ~2.5 cm) is either filled with CSF in the acute state or scar tissues in chronic state (Jacobs et al., 2001; Soltanian-Zadeh et al., 2003). Based on these, we modeled the skull defects as follows: (1) 10-cm diameter defect modeled as a titanium plate; (2) 10-cm diameter defect modeled as an acrylic plate; (3) 2.5-cm diameter defect modeled as acute injury (filled with the CSF); (4) 2.5-cm diameter defect modeled as chronic scar tissue. Note in this paper we use “defect” to refer to the openings on the skull that are either implanted with a plate or filled with CSF or scar tissue. The defect was first modeled as a cylinder and placed manually in ScanCAD (Simpleware Ltd., Exeter, UK) with the center above the right primary motor cortex and normal to the local scalp surface. The intersection of the cylinder and the skull segmentation was then classified as the defect and was assigned a different electrical conductivity when computing the lead field (see the next subsection).

### Optimized HD-tDCS

A customized version of the open-source software ROAST (Huang et al., 2019; Huang, 2020) was used to solve for the forward model (also known as the lead field) needed for optimized HD-tDCS (Dmochowski et al., 2011). Specifically, the customized ROAST takes the segmentation of six tissues from the MIDA model. 74 electrodes of 6 mm radius following international 10–10 convention (Klem et al., 1999) were placed on the scalp. To avoid complications in automatically placing electrodes near or behind the ear-lobes, we omitted positions TP9 and TP10. The entire volume was then discretized into a finite element mesh, and the forward problem was solved for each bipolar montage with electrode Iz as the reference. See Huang et al., (2013, 2019) for more details. If the skull defect was added into the model, then in total seven tissues were modeled. This entire process was done fully automated in the customized ROAST. Default conductivities in ROAST were assigned to the six tissues (in S/m: white matter – 0.126, gray matter – 0.276, CSF – 1.65, skull – 0.01, scalp – 0.465, air cavities – 2.5 × 10^−14^; Huang et al., 2013), and skull defects were assigned with the following conductivities (in S/m): (1) titanium – 7.4 × 10^5^; (2) acrylic – 2.0 × 10^−13^; (3) CSF – 1.65; (4) scar – 0.34 (Datta et al., 2010). Optimized HD-tDCS was performed to stimulate the right primary motor cortex (MNI coordinates x = 48, y = −8, z = 50) below the skull defect with highest possible intensity and stimulating current on the scalp not exceeding the safety limit of 2 mA (Dmochowski et al., 2013). This was done also in ROAST using the “roast_target()” function. The achieved electric field magnitude at the target location was recorded for each skull defect model and the normal head model (without any skull defect). We released the customized version of ROAST at the Github repository (Huang, 2020).

### Comparison between models and montages

For all the skull defect models and the normal model, we also simulated the electric field distribution for two un-optimized electrode montages in ROAST: M1-SO and 4×1. For the M1-SO montage, conventional pad electrodes were used with the anode placed on top of the right primary cortex (electrode C4) and the cathode placed at Fp1. For the 4×1 montage, 6-mm radius anode was placed at C4, with cathodes surrounding at FC2, FC6, CP2, and CP6. In both cases, the total injected current was 2 mA.

We compared the achieved electric field magnitude at the right primary motor cortex across different models (4 skull defect models and the normal model) and montages (optimized, M1-SO, 4×1). To test the robustness of optimized HD-tDCS and sample more data points from the models to compare, we also shifted the target location, re-ran the optimization, and compared the achieved field magnitude across models and montages. We shifted the target location in four directions: anterior by 2 cm, posterior by 2 cm, left by 2 cm, and right by 1 cm (instead of 2 cm which is out of the brain). Mann–Whitney U test was used to assess the significance of the difference between models.

## Results

### Construction of head and skull lesion models

**Figure 1:**
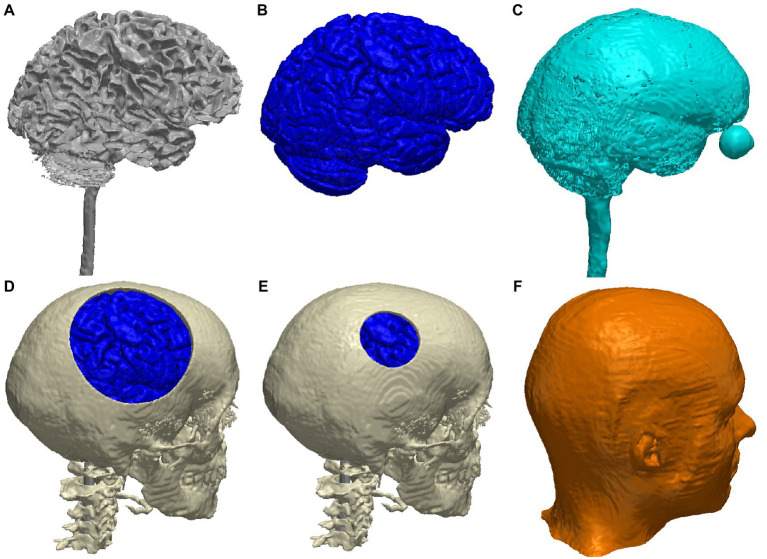
3D renderings of the major tissue types in the MIDA head model: **(A)** white matter; **(B)** gray matter; **(C)** CSF; **(D)** skull with a large defect (10-cm diameter, gray matter can be seen through the defect); **(E)** skull with a small defect (2.5-cm diameter); **(F)** scalp.

**Figure 2:**
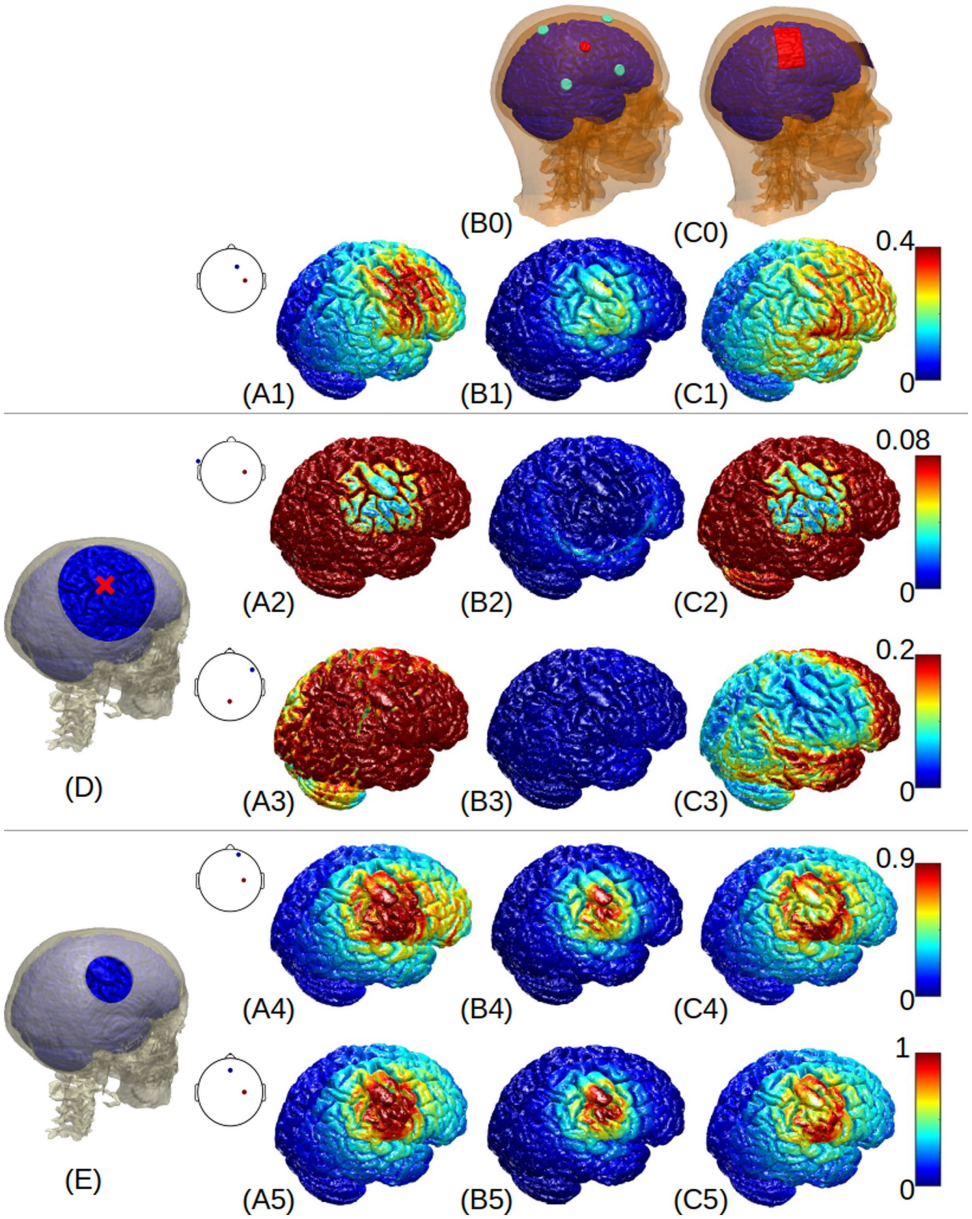
3D renderings of electric field around the right primary motor cortex (indicated by the red cross in **(D)**) generated by the normal-anatomy model **(A1-C1)**, large skull defect with titanium conductivity **(A2-C2)**, large defect with acrylic conductivity **(A3-C3)**, small defect with CSF conductivity **(A4-C4)**, and small defect with scar tissue **(A5-C5)**. Column **(A)** shows the results from optimized HD-tDCS with the optimal montages shown as insets at each panel; Columns **(B)** and **(C)** show the results from 4×1 and M1-SO montages, respectively. The skull defects are shown in panels **(D)** and **(E)**. A colormap for each row is shown on the right side, with a unit of V/m.

The merged segmentation of the head tissues from the MIDA model, with the skull defects, is shown in Figure 1. Note that we centered the skull defect right above the right primary motor cortex (Figure 2D).

### Optimized HD-tDCS

**Figure 3:**
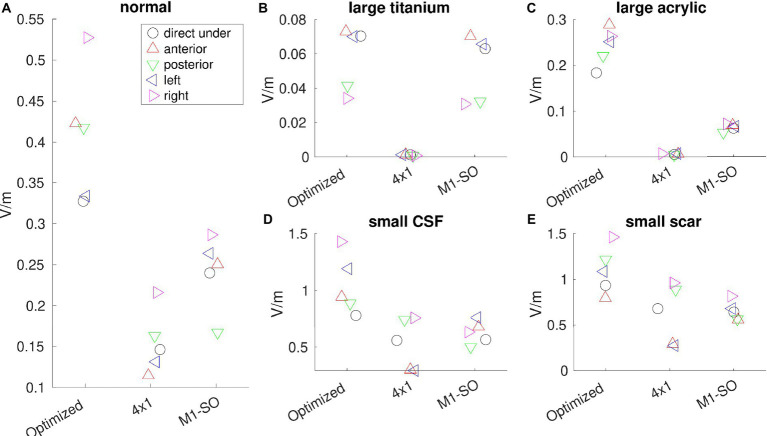
Electric field (V/m) read out from each model under different montages (optimized, 4×1, and M1-SO). **(A)** model with normal anatomy; **(B)** large skull defect with titanium conductivity; **(C)** large defect with acrylic conductivity; **(D)** small defect with CSF conductivity; **(E)** small defect with scar tissue. Electric fields are read out from the right primary motor cortex (circle marker) which is directly under the skull defect with MNI coordinates (48, −8, 50), as well as from locations anterior (up-pointing triangle), posterior (down-pointing triangle), left (left-pointing triangle), or right (right-pointing triangle) to the right primary motor cortex.

Figure 3 shows the electric field from each model under different montages. It is notable that optimized stimulation always boosts the intensity at the target compared to un-optimized montages, no matter whether the skull has a defect or not. Specifically, for the location directly under the skull defect [circle marker, MNI coordinates (48, −8, 50)], optimized stimulation in a normal-skull model boosts the stimulation intensity at the target by 0.18 V/m (4×1 montage) and 0.09 V/m (M1-SO montage). For the large defect with a titanium plate, the increase is 0.07 V/m for 4×1 montage and 0.01 V/m for M1-SO montage. For the large defect with an acrylic plate, the increase is 0.18 V/m (4×1) and 0.12 V/m (M1-SO). For the small defect with CSF, the increase is 0.22 V/m for both 4×1 and M1-SO montages. For the small defect with scar tissue, the increase is 0.25 V/m (4×1) and 0.29 V/m (M1-SO).

### Comparison between models and montages

Skull defects change the stimulation intensity. As shown in Figure 3, for the optimized montage, electric field at the right primary motor cortex decreases from 0.33 V/m to 0.07 V/m for the large titanium plate, to 0.18 V/m for the large acrylic plate, and increases to 0.78 V/m for the small defect with CSF, and to 0.93 V/m for the small defect with scar tissue. Mann–Whitney U test shows that the changes in stimulation intensities by skull defects are significant for all the four skull-defect models (*p* < 0.01).

Figure 2 visualizes the electric field distribution. Again we see that, compared to the normal anatomy, large skull defects with titanium or acrylic plates reduce the electric field at the right primary motor cortex, while small defects with CSF or scar tissue increase the electric field. This is true for all the montages. The small skull defect seems to increase the focality of the stimulation (Figures 2A4–C4 A5–C5), while the large defect seems to blur the stimulation focality (Figures 2A2–C2 A3–C3). The large titanium plate shunts away electric current (Figures 2A2 C2), and the large acrylic plate insulates the current (Figure 2B3).

When considering all the five locations (target and four shifted locations, Figure 3), Mann–Whitney U test showed that the boost by optimized stimulation is significant for all the cases (*p* < 0.05), except two scenarios: (1) for large defect with a titanium plate, the difference in stimulation intensity is not significant between optimized montage and the conventional M1-SO montage (*p* = 0.42, Figure 3B); (2) for small defect with scar tissue, optimized montage does not significantly increase the stimulation from 4×1 montage (*p* = 0.06, Figure 3E).

## Discussion

To the best of our knowledge, this work is the first computational study to compare optimized HD-tDCS with conventional electrode montages on a head model with a skull defect. Existing work in the literature mostly focus on how the forward models of electroencephalogram (EEG) is affected by skull defects (Lau et al., 2016), skull segmentation (Lanfer et al., 2012), skull conductivity (Antonakakis et al., 2019b), and skull suture (McCann and Beltrachini, 2022). The only work we found that studied how skull defects affect optimized tDCS is Antonakakis et al. (2019a), but it only looked at small burr holes on the skull instead of skull plates. Our previous work (Datta et al., 2010) studied how skull defects affect the current flow but did not compare between un-optimized and optimized montage stimulating the cortex under the defect. Here we investigated how different sizes and conductivities of skull defects affect the current flow on the cortex beneath the defects, for both un-optimized and optimized stimulation. We found that large defects with titanium or acrylic plates significantly reduces the electric current reaching the target area beneath the defect by about 80%, while small defects with CSF or scar tissue significantly increases the stimulation by about 200%. Optimization always increases the stimulation intensities at the target area, no matter if the skull has a defect or not, even though this increase is not significant when a large defect with titanium plate or a small defect with scar tissue is present on the skull.

From the safety standpoint, the increase in electric field by 200% does not raise any potential theoretical safety issue. Using epicranial electrode stimulation in rats, Liebetanz and colleagues demonstrated that the threshold for tissue damage is at least two orders of magnitude away from the scalp charge density applied in humans (Liebetanz et al., 2009). Further, one may expect similar electric field deviation even in intact anatomy across individual heads (Datta et al., 2012). If the study objective requires maintaining the same electric field magnitude, a simple abating strategy would be to reduce the scalp injected current in proportion to the increase. Finally, optimized HD-tDCS has already been safely delivered to stroke subjects including cases where the cortical electric field was found to triple in comparison to conventional tDCS delivery (Dmochowski et al., 2013; Richardson et al., 2015).

To address the 80% decrement, a compelling clinical strategy would be to increase the scalp injected current. Higher intensity tDCS (i.e., delivery of 3–4 mA scalp current) has been recently shown to be safe (Workman et al., 2020; Hsu et al., 2023). While doubling the scalp intensity would only cover for the 50% decrement, what is clear is that scaling scalp current offers an option to get closer to what may be considered as “efficacious dose.” Ultimately, clinicians would have to make the decision based on the potential risk–benefit, as tDCS may be one of the few interventions available considering the high vulnerability of patients with skull defects and plates.

Note that the strategies above are only general guidelines on tDCS on patients with skull defects or plates, as they are only based on the results from the single subject model we obtained here. Future modeling studies leveraging individualized geometry of the skull defects/plates obtained from patients MRI and CT scans will be needed to further provide personalized guidelines and plans on improving the outcomes from tDCS therapy.

Besides the average of 0.3 V/m increase of stimulation intensities at the target compared to un-optimized montages, the utility of optimization is best exemplified by targeting a region directly under the large skull acrylic plate. In general, the very low conductivity of acrylic makes it difficult to deliver meaningful electric field intensity directly underneath the plate (Datta et al., 2010). However, using optimized HD-tDCS, we are able to obtain ~0.18 V/m and as mentioned above, potentially deliver an efficacious dose by a simple scaling of scalp current. This is in stark contrast to the traditional montages, where the very low induced target electric field makes pursuing them unworthy.

There are some limitations of this work. First, we only modeled the skull defect at one single location which is mostly motivated by the clinical scenario (Fatima et al., 2019; Lambride et al., 2020). However, the same physics and optimization algorithm apply to defects at other locations on the skull. Second, we simplified the shape of the defect, while in reality the defect could have a complicated shape. Future work will collect image data from patients with skull defects to model the actual geometry of skull defects. Third, only one individual head was modeled. Considering inter-individual variability, future work will repeat the modeling process on more heads with skull defects to confirm if the results are replicable on other individual heads. Lastly, all the results were obtained from computational models, which need to be confirmed by experimental measurements following previous methodology (Huang et al., 2017).

## Data availability statement

The original contributions presented in the study are included in the article/supplementary material, further inquiries can be directed to the corresponding author.

## Author contributions

AG contributes to build the initial head model and draft of the manuscript. DT contributes to build the initial head model and editing of the manuscript. AD contributes to the concept of this work, and editing of the manuscript. YH contributes to the concept of this work, implementation of the model optimization, analysis of results, and editing of the manuscript. All authors contributed to the article and approved the submitted version.
